# Directed Inward Migration of S‐Vacancy in Bi_2_S_3_ QDs for Selective Photocatalytic CO_2_ to CH_3_OH

**DOI:** 10.1002/advs.202406925

**Published:** 2025-01-09

**Authors:** Jing Wang, Wenlei Wang, Yao Deng, Zhen Zhang, Hui Wang, Yiqiang Wu

**Affiliations:** ^1^ College of Materials Science and Engineering National and Local Joint Engineering Research Center for Green Processing Technology of Agricultural and Forestry Biomass Central South University of Forestry and Technology Changsha 410004 China; ^2^ College of Chemistry and Chemical Engineering National Forestry and Grassland Administration Bioethanol Research Center College of Chemistry and Chemical Engineering Central South University of Forestry and Technology Changsha 410004 China

**Keywords:** CO_2_, CH_3_OH, directed inward migration, S Vacancy, selective photocatalytic

## Abstract

The directional migration of S‐vacancy is beneficial to the separation of photogenerated carriers and the transition of electrons in semiconductors. In this study, Bi_x_/Bi_2−x_S_y_@carboxylic‐cellulose (CC) photocatalyst with bionic chloroplast structure is obtained by electron beam irradiation to induce S‐vacancy in Bi_2_S_3_@CC. The results of CO_2_ photoreduction experiments demonstrate that the reduction rate of CO_2_ to CH_3_OH by Bi_x_/Bi_2‒x_S_2.89_@CC‐450 samples is 10.74 µmol·g^−1^·h^−1^, and the selectivity is 92.82%. The results show that the inward migration behavior of the borderline S‐vacancy (b‐S_v_) induces the redistribution of electrons in Bi_x_/Bi_2−x_S_y_@CC. The Bi° clusters in Bi_x_/Bi_2−x_S_y_@CC is conducive to adsorb CO_2_, and the internal S‐vacancy (i‐S_v_) is conducive to adsorb CH_3_OH, which accelerate the transfer of gas‐phase products to realize the controllable conversion of CO_2_ and photoreduction products at the gas–liquid–solid three‐phase interface. This study provides a new idea for the development and utilization of green photocatalysts in clean energy.

## Graphical abstract

The Bi_x_/Bi_2−x_S_y_@CC photocatalyst with bionic chloroplast structure is synthesized by inducing S‐vacancies through electron beam irradiation. The study demonstrates that Bi_x_/Bi_2−x_S_y_@CC reduce CO_2_ to CH_3_OH with high efficiency and selectivity. The inward migration of S‐vacancy redistributes electrons, promoting gas‐phase product transfer and controllable CO_2_ conversion at the gas–liquid–solid interface, advancing green photocatalyst development for clean energy.

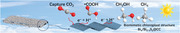

## 1 Introduction

Since the Industrial Revolution, the amount of greenhouse gases emitted into the atmosphere by human beings has been increasing year by year, and a series of issues caused by the greenhouse effect in the atmosphere have aroused the concern of countries worldwide.^[^ ^1^, ^2^, ^3^ ^]^ Currently, the simulation of photosynthesis in nature and the production of valuable chemicals using carbon dioxide as a carbon source are the fundamental ways to address the energy crisis and environmental pollution issues currently faced by humanity.^[^ ^4^, ^5^ ^]^ As a relatively green and environmentally friendly new technology, photocatalytic technology stands out among many carbon sink technologies.^[^ ^6^ ^]^ Currently, the main bottlenecks of photocatalytic carbon sink technology are mainly lie in the following aspects. First, the photocatalysts have low light collection capacity and energy conversion efficiency, which makes it more challenging to find appropriate photocatalysts.^[^ ^7^ ^]^ Second, the selectivity of photoreduction products is low, and the reaction products are chaotic and complex.^[^ ^8^ ^]^ Finally, the structure–property relationship, reaction mechanism, and directional transfer mechanism of products in the photocatalytic reduction of CO_2_ have not yet been clarified, which makes it challenging to realize industrial applications.^[^ ^9^ ^]^ Therefore, it is relevant to design environmentally friendly and efficient photocatalysts to realize high‐value conversion of CO_2_.

The ability of photocatalysts to drive CO_2_ conversion mainly depends on three key factors: light capture, carrier separation, and surface reaction.^[^ ^10^, ^11^ ^]^ To address the above technical bottlenecks, researchers have made many efforts. From loading metals to tuning the size of metal cocatalysts, all are aimed at addressing the issue of photocatalytic efficiency.^[^ ^12^ ^]^ Bi_2_S_3_ is a narrow bandgap (1.3–1.7 eV) semiconductor photocatalyst with substantially high utilization of visible light.^[^ ^13^ ^]^ However, the single‐component Bi_2_S_3_ photocatalyst has the disadvantages of severe photocorrosion and a high charge complexation rate, which significantly limit the visible light catalytic activity.^[^ ^14^ ^]^ Therefore, the Bi_2_S_3_ composite photocatalytic material has high visible light utilization while effectively suppressing electron–hole complexation, thus improving its photocatalytic activity. This shows a wide range of applications in the research field of photocatalytic reduction of CO_2_.^[^ ^15^, ^16^ ^]^ Xi et al. prepared a network BiOBr/Bi_2_S_3_ nanoarray heterojunction photocatalyst with hierarchical pores and oxygen vacancies and applied it to wide CO_2_ photoreduction. The results reveal that this reticular Bi_2_S_3_ nanoarray grown on BiOBr nanoplates can lead to the conversion of CO_2_ to CO with a precipitation rate of 103.5 µmol·g^−1^·h^−1^ and a selectivity of up to 90.1% under light.^[^ ^17^ ^]^ Alkanad et al. constructed a Bi_2_S_3_/TiO_2_/MoS_2_ S‐Scheme heterogeneous photocatalyst. The results indicate that Bi_2_S_3_/TiO_2_/MoS_2_ can regulate electron flow direction and REDOX potential and selectively photocatalyze the reduction of CO_2_.^[^ ^18^ ^]^

The vacancy in the photocatalyst usually promotes the rapid separation of the photogenerated electron–hole pair by changing the electron distribution on the surface of the material to realize the effective use of the photogenerated carrier.^[^ ^19^ ^]^ The zero‐valent metal in the photocatalyst can often act as a charge transfer “bridge” and electron donor to promote the directional transfer of charge carriers in the photoreaction.^[^ ^20^ ^]^ The synergistic effect of vacancy and zero‐valent metal in the material benefits for photocatalytic reactions. Li et al. reported an O_V_‐BiOBr/Ni_2_P heterojunction composite with reinforcing chemical force. They pointed out that specific concentration of O_V_ in the O_V_‐BiOBr/Ni_2_P heterojunction can effectively promote carrier separation. The in‐situ precipitation of Bi^0^ metal offers more active sites for hydrogen evolution reactions.^[^ ^21^ ^]^ Zhang et al. synthesized Bi_2_MoO_6_ with Bi vacancies and Bi^0^ by a superficial chemical reduction method. The results reveal that the vacancy helps increase the band gap of the material to facilitate photogenerated electron–hole pair separation, as Bi° can act as an electron trap to accelerate charge transfer through solid interaction with Bi_2_‐δMoO_6_.^[^ ^22^ ^]^ S‐vacancy refers to the missing positions of S atoms in the lattice, creating lattice defects. These defects play a crucial role in enhancing photocatalytic performance. Compared to simple Bi_2_S_3_ photocatalysts, Bi_2_S_3_ photocatalysts with S‐vacancy significantly improve photocatalytic performance by enhancing light absorption, promoting the separation of photogenerated charge carriers, increasing surface active sites, accelerating interfacial charge transfer, tuning the electronic structure, and improving chemical stability. These advantages make Bi_2_S_3_ with S‐vacancy highly promising for photocatalytic applications, demonstrating higher efficiency in areas such as environmental pollutant degradation and photocatalytic water splitting for hydrogen production. Electron beam irradiation is a new processing technology. The principle is to use the electron rays accelerated in the high‐voltage electric field to irradiate the substance through the interaction of high‐energy electrons with the substance to ionize and stimulate the molecules of various substances, thereby triggering chemical reactions to improve the performance of the material. The interaction between electrons and matter is to break the chemical bond through the formation of new chemical reactions, the decomposition of organic matter, or the dislocation of atoms.^[^ ^23^, ^24^, ^25^ ^]^ Therefore, electron beam irradiation is expected to be used in material defect engineering.

Carboxylated cellulose is a water‐soluble polymer derived from natural cellulose through carboxymethylation modification.^[^ ^26^ ^]^ Due to its unique chemical structure and physical properties, it offers significant advantages across various fields, particularly in photocatalytic composite materials. The addition of carboxylated cellulose can enhance the overall performance of these materials. Carboxylated cellulose contains numerous carboxyl (‒COOH) functional groups, which exhibit strong hydrophilicity, allowing it to disperse and stabilize well in water or other polar solvents. When combined with other photocatalysts, such as TiO_2_, ZnO, or Fe_2_O_3_, carboxylated cellulose can effectively improve the dispersion of the photocatalyst, preventing the accumulation of nanoparticles, thus maintaining a high surface area and photocatalytic activity. The surface of carboxylated cellulose is rich in carboxyl groups, which can interact with pollutants or target reactants through complexation or electrostatic adsorption, enhancing the photocatalyst's ability to adsorb pollutants. This increased adsorption facilitates more excellent contact between the contaminants and the surface of the photocatalyst, thereby promoting the photocatalytic degradation reaction. Introducing carboxylated cellulose can effectively alter the surface chemical properties of the photocatalyst. For instance, carboxyl groups can adjust the hydrophilicity or hydrophobicity of the photocatalyst, increasing its surface reactivity. These carboxyl functional groups can interact with the active sites on the surface of the photocatalyst, influencing electron transfer processes and promoting both oxidation and reduction reactions during photocatalysis. Furthermore, carboxylated cellulose can significantly enhance the long‐term stability of photocatalysts. Photocatalysts are prone to deactivation during prolonged light exposure, especially in aqueous or organic solutions where nanoparticles may agglomerate or undergo structural changes. Due to its excellent dispersing and stabilizing properties, carboxylated cellulose helps protect the photocatalyst particles from accumulation and morphological changes, extending their operational lifespan. In summary, carboxylated cellulose offers notable advantages in photocatalytic composite materials, improving the dispersibility, adsorption properties, photocatalytic efficiency, and mechanical strength of photocatalysts.

Here, stable Bi_2_S_3_@CC photocatalysts were constructed based on carboxylic cellulose (CC). Electron beam irradiation induced the generation and directional migration of S‐vacancy in Bi_2_S_3_@CC to obtain Bi_x_/Bi_2‒x_S_y_@CC photocatalysts with a bionic chloroplast structure. Finally, the prepared Bi_x_/Bi_2‒x_S_y_@CC photocatalyst was used for the photoreduction of CO_2_ to prepare CH_3_OH. The orientation migration behavior of S‐vacancy in Bi_x_/Bi_2‒x_S_y_@CC was tracked by transmission electron microscopy (TEM). Density functional theory (DFT) was applied to investigate the effect of directional transfer of S‐vacancy in Bi_x_/Bi_2‒x_S_y_@CC on the surface charge distribution. The inward migration behavior of S‐vacancy in Bi_x_/Bi_2‒x_S_y_@CC was clarified by photoelectronic tests and in situ infrared for the law and mechanism of photocatalytic reduction of CO_2_ to CH_3_OH. This study provides new ideas for applying cellulose‐based bionic chloroplast‐structured photocatalysts in CO_2_ capture and conversion.

## 2 Results and Discussion

### 2.1 Characterization of the Prepared Samples

**Figure 1:**
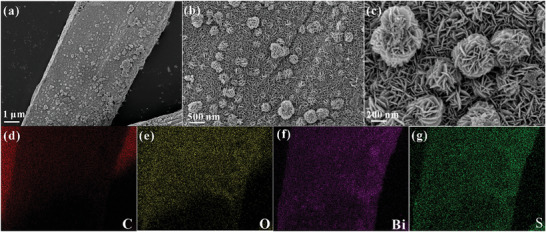
a,b) SEM and EDS elemental mappings of c) C, d) O, e) Bi, f) S in Bi_x_/Bi_2‒x_S_2.89_@CC‐450 samples.

**Figure 2:**
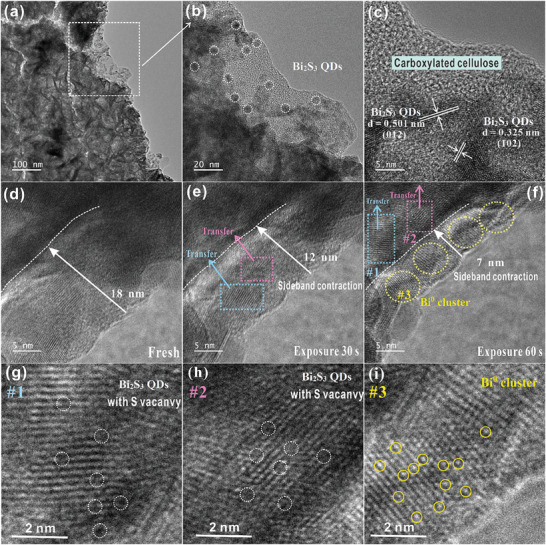
TEM diagram of a–c) Bi_x_/Bi_2‒x_S_2.89_@CC‐450; HRTEM images of Bi_2_S_3_@CC with different electron beam residence times: d) 0 s, e) 30 s, f) 60 s; high‐resolution images of g) #1, h) #2, and i) #3 in (f).

SEM was used to study the microscopic morphology of the prepared samples. As shown in Figure S1 (Supporting Information), the pure CC is a fiber bundle consisting of multiple nanofibrillar filaments with smooth surfaces and a diameter of ≈5 µm. **Figure** **1**a–c shows the SEM images of the Bi_x_/Bi_2‒x_S_2.89_@CC‐450 samples, and it can be observed that the flaky or flowery clusters of Bi_2_S_3_ QDs are uniformly loaded on the surface of the CC, which is comparable with the chloroplasts that are similar. EDX‐mapping results demonstrated uniform dispersion of C, O, Bi, and S elements, suggesting the samples were successfully prepared (Figure 1d–g). **Figure** **2**a–c shows the transmission electron microscopy (TEM) images of the Bi_x_/Bi_2‒x_S_2.89_@CC‐450 sample, resulting in the regions showing no apparent lattice as amorphous CC. By contrast, the lattice spacings of 0.501 and 0.325 nm correspond to the (012) and (102) crystal planes of Bi_2_S_3_ QDs, respectively. The Bi_2_S_3_ QDs are uniformly loaded on the CC surface and have a size of ≈5 nm. To understand the effect of electron beam irradiation on the initial sample (Bi_2_S_3_@CC), TEM was used to simulate the process of electron beam irradiation. As shown in Figure 2d, Bi_2_S_3_@CC without electron beam irradiation presents a sideband region of ≈18 nm, in which a lattice belonging to Bi_2_S_3_ QDs can be observed. After 30 s of irradiation with an electron beam, the sideband region in Figure 2d started to shrink inward to ≈12 nm. By contrast, it can be seen the appearance of Bi^0^ in regions #1 and #2 (Figure 2e). The irradiation time of the electron beam is extended to 60 s, and the sideband region is further shrunk to 7 nm (Figure 2f). Region #1 and #2 gradually migrate into the borderline and generate Bi^0^ clusters at region #3 (Figure 2g,h). To further investigate this exciting migration phenomenon, high‐resolution analysis of regions #1, #2, and #3 was performed. The results indicate that atom deletions can be observed in regions #1 and #2. By contrast, apparent Bi^0^ clusters can be observed in region #3, suggesting that electron beam irradiation can be used to induce vacancy generation and migration in the material. Moreover, High Angle Annular Dark Field‐STEM (HAADF‐STEM) images of the Bi_x_/Bi_2‒x_S_2.89_@CC‐450 sample are shown in Figure S2 (Supporting Information). The presence of Bi⁰ clusters and S‐vacancy can be observed.

**Figure 3:**
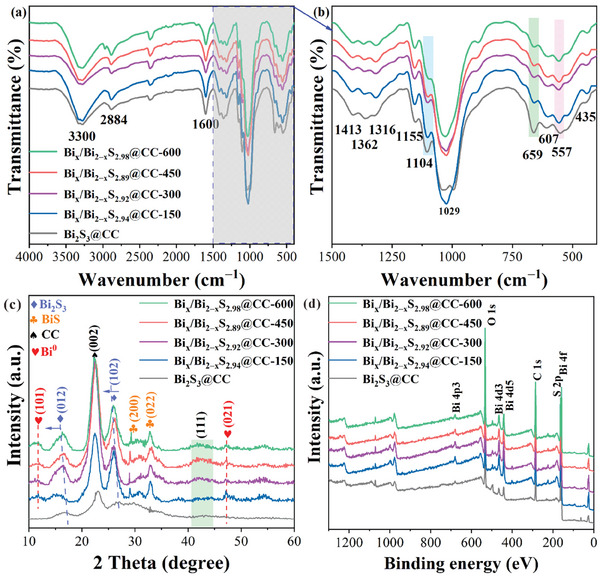
a,b) FT‐IR, c) XRD, and d) XPS full spectrum of each sample.

FT‐IR spectroscopy is used to obtain chemical bond and functional group information on the sample. As shown in **Figure** **3**a,b, the peaks at 3300 and 1362 cm^−1^ are classified as the stretching and bending vibrations of –OH on the glucose unit, respectively.^[^ ^27^ ^]^ The characteristic peaks at 2884 and 1316 cm^−1^ are classified as the C─H tensile vibration of methylene and the oscillating vibration of CH_2_, respectively.^[^ ^28^ ^]^ The characteristic peaks at 1600 and 1413 cm^−1^ are the asymmetric vibrations of C = O on the carboxyl group and the stretching vibration of ─COO─, respectively.^[^ ^29^ ^]^ The peaks at 1155, 1104, and 1029 cm^−1^ correspond to the antisymmetric stretching vibrations of C─O─C in the pyran ring.^[^ ^30^ ^]^ Notably, the intensity of the characteristic peak at 1104 cm^−1^ decreases with the increase of electron beam irradiation intensity, which may be attributed to the migration of Bi_2_S_2‒x_ with S‐vacancy, which makes the electrons on C─O─C biased toward the electron‐deficient Bi_2_S_2‒ x_, resulting in the weakening of the stretching vibration of C─O─C.^[^ ^31^ ^]^ In addition, peaks at 659 and 607 cm^−1^ are attributed to the flexural and tensile vibrations of the Bi─S bond in Bi_2_S_3_ QDs, respectively.^[^ ^32^ ^]^ The peaks at 557 and 435 cm^−1^ are classified as Bi─O bond flexural and tensile vibrations, respectively, suggesting that Bi_2_S_3_ and CC are bonded in a chemical bond form.^[^ ^33^ ^]^ Notably, the intensity of the characteristic peak at 659 cm^−1^ also decreases with the increase in electron beam irradiation intensity, which is the result of the Bi─S bond breaking.

The phase composition and crystal structure of the prepared catalyst were determined by XRD. As shown in Figure 3c, the solid characteristic diffraction peak at 2θ = 22.41° of the prepared sample can be attributed to the (002) crystal face of CC. The characteristic diffraction peaks at 2θ = 16.77 and 26.90° correspond to the (012) and (102) crystal faces of Bi_2_S_3_, respectively. Notably, after electron beam irradiation, the characteristic diffraction peaks corresponding to the (012) and (102) crystal faces of Bi_x_/Bi_2‒x_S_y_@CC are shifted to a smaller angle, which is due to the lattice distortion caused by the reduction of part of Bi^0^ after electron beam irradiation bombardment of Bi_2_S_3_ QDs, resulting in larger cell volume and crystal face spacing. Meanwhile, the characteristic diffraction peaks of Bi_x_/Bi_2‒x_S_y_@CC at 2θ = 29.24 and 32.79° correspond to the (200) and (022) crystal faces of BiS, respectively. By contrast, the characteristic peaks of Bi_2_S_3_@CC do not appear at the same position, which is the result of Bi^3+^ being reduced by electron beam. In addition, the characteristic diffraction peaks of Bi_x_/Bi_2‒x_S_y_@CC at 2θ = 11.85 and 46.83° are classified as (101) and (021) of Bi^0^ of the hexagonal crystal system, and there are no apparent characteristic peaks of Bi_2_S_3_@CC at the same position, which further proves that Bi^0^ clusters can be successfully obtained by electron beam irradiation. Moreover, the diffraction peak at 2θ = 44.5° corresponds to the (111) crystal plane of the graphite phase in the CC sample. Due to the overall weaker intensity of the characteristic diffraction peaks in the Bi_2_S_3_@CC sample, the diffraction peak at 2θ = 44.5° is less prominent than other samples.

**Figure 4:**
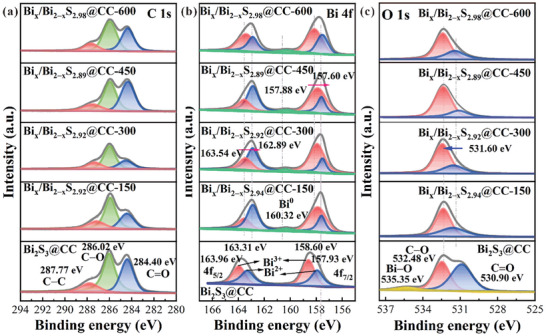
XPS spectra of elements a) C, b) Bi, and c) O of each sample.

XPS is used to obtain information about the element composition, content, chemical state, molecular structure, and chemical bonds of the prepared samples. Figure 3d shows the full spectrum of each sample, and the characteristic peaks containing C, O, Bi, and S elements can be observed, which is consistent with the EDX results. **Figure** **4**a shows the XPS spectrum of the C element. It can be observed that the binding energy at 287.77, 286.02, and 284.40 eV is directed to the C–C, C─O, and C═O bonds in CC, respectively. The XPS spectrum of the Bi element is shown in Figure 4b. It can be observed that the characteristic peaks of the Bi_2_S_3_@CC sample at binding energies of 163.96 and 158.60 eV are classified as 4f_5/2_ and 4f_7/2_ of Bi^3+^, respectively. The binding energies of 163.31 and 157.93 eV are classified as 4f_5/2_ and 4f_7/2_ of Bi^2+^, respectively, consistent with XRD results. After electron beam irradiation, the characteristic peaks at the binding energy of 160.32 eV belong to Bi^0^ in Bi_x_/Bi_2‒x_S_y_@CC, and the characteristic peaks at the binding energy of 162.89 and 157.60 eV belong to 4f_5/2_ and 4f_7/2_ in Bi^2+^. Moreover, the characteristic peak of Bi^3+^ moved to the lower binding energy, suggesting that Bi^3+^ in the Bi_2_S_3_@CC sample obtained electrons and underwent a reduction reaction. The XPS spectrum of S element is shown in Figure S3 (Supporting Information), and the characteristic peaks at binding energies of 163.91 and 163.19 eV in the Bi_2_S_3_@CC sample are classified as S 2p_1/2_ and 2p_3/2_, respectively. Compared to the Bi_2_S_3_@CC, the XPS peaks of the S element in the Bi_x_/Bi_2‒x_S_y_@CC sample shift to lower binding energies. On the one hand, this is likely due to the electron beam irradiation generating a large number of free electrons in the material, which can accumulate around the sulfur atoms, increasing the local electron density of the sulfur. As S atoms gain more electrons, the binding energy detected by XPS decreases accordingly. On the other hand, the electron beam irradiation may break chemical bonds in the composite material, causing lattice distortion and the formation of S‐vacancy. These changes lead to a redistribution of the electron cloud around the sulfur atoms, further lowering the binding energy of sulfur. Since XPS is typically used to characterize surface defects in materials, it can be concluded that surface sulfur vacancies are present in the Bi_x_/Bi_2‒x_S_y_@CC sample. The XPS spectrum of element O is shown in Figure 4c, and the characteristic peaks at binding energies of 525.35, 532.48, and 530.90 eV correspond to the Bi–O, C─O, and C═O bonds in Bi_2_S_3_@CC, respectively. After electron beam irradiation, the characteristic peaks related to the Bi–O bond in Bi_x_/Bi_2‒x_S_y_@CC disappear, and the binding energy of the C═O bond moves to higher energy, suggesting that a part of the Bi–O bond is interrupted to form Bi^0^ breaks and that the binding mode between Bi_2_S_3_ QDs and CC is in the form of a chemical bond, which is consistent with the results of FT‐IR. The contents of each element in the substance were calculated according to XPS, and the results are shown in Table S1 (Supporting Information). When the electron beam irradiation intensity is 450 KGy, the contents of Bi^0^, Bi^n+^, S, C, and O in Bi_x_/Bi_2‒x_S_2.89_@CC‐450 are ≈1.42, 17.28, 24.84, 38.46, and 18.00%, respectively. However, when the electron beam irradiation intensity is further increased to 600 KGy, the contents of Bi^0^, Bi^n+^, S, C, and O in Bi_x_/Bi_2‒x_S_2.98_@CC‐600 are ≈1.29, 17.85, 26.63, 38.98, and 15.25%, respectively. It is noteworthy that although electron beam irradiation can induce the generation of Bi⁰ and the migration of S vacancies in Bi_x_/Bi_2‐x_S_y_@CC, a strong electron beam can instantly disrupt the sample structure, reducing the effectiveness of electron beam reduction and leading to a decrease in the contents of Bi⁰ and S‐vacancy in the sample. Therefore, it is essential to select an appropriate electron beam irradiation intensity when regulating sample defects.

### 2.2 Performances of the Prepared Catalysts

**Figure 5:**
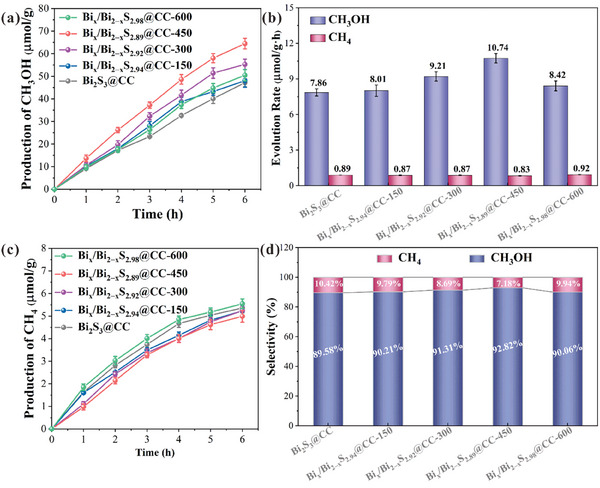
a) Time courses of photocatalytic activity for CH_3_OH on different photocatalysts. b) Evolution rates of CH_4_ and CH_3_OH on different photocatalysts. c) Time courses of photocatalytic activity for CH_4_ on different photocatalysts. d) Selectivity of CO_2_ reduction products on different photocatalysts.

Without any photosensitizer or sacrificial agent, the photoreduction activity of the bionic chloroplast photocatalyst was measured. The results revealed that CH_3_OH and CH_4_ were detected simultaneously in the products (**Figure** **5**). Bi_2_S_3_@CC showed the slowest reduction rate of CO_2_ to CH_3_OH (7.86 µmol·g^−1^·h^−1^), the fastest reduction rate of CO_2_ to CH_4_ (0.89 µmol·g^−1^·h^−1^), and the selectivity for methanol was only 89.58%. The Bi_x_/Bi_2‒x_S_2.89_@CC‐450 sample showed the fastest reduction rate of CO_2_ to CH_3_OH (10.74 µmol·g^−1^·h^−1^), the slowest reduction rate of CO_2_ to CH_4_ (0.83 µmol·g^−1^·h^−1^), and the selectivity for methanol was up to 92.82%. Calculate the photon conversion efficiency (PCE) and apparent quantum efficiency (AQE) of the photocatalyst to evaluate the actual photon utilization efficiency of the photocatalyst under specific conditions. The calculation formula is as follows.

(1) $$PCE=\frac{\mathrm{Energy}\;\mathrm{for}\;CH_{3}\mathrm{OH}\;\mathrm{Generation}}{\mathrm{Input}\;\mathrm{Light}\;\mathrm{Energy}}\;\times 100\%$$

(2) $$\begin{array}{ccc} AQE & = & \frac{\left(\mathrm{Number}\;\mathrm{of}\;\mathrm{Product}\;CH_{3}\mathrm{OH}\right)\times \left(\mathrm{Number}\;\mathrm{of}\;\mathrm{Electrons}\right)}{\left(\mathrm{Number}\;\mathrm{of}\;\mathrm{Incident}\;\mathrm{Photons}\right)}\;\\ & & \times 100\% \end{array}$$

The PCE and AQE of the Bi_x_/Bi_2‒x_S_2.89_@CC‐450 sample for CO₂ photoreduction under 300 W xenon lamp irradiation over 6 h was 0.0202 and 0.0509%, respectively. Compared with similar catalysts reported in the literature (Table S2, Supporting Information), the Bi_x_/Bi_2‒x_S_2.89_@CC‐450 demonstrates higher competitiveness in the photocatalytic reduction of CO₂ to methanol (refs. [S1–S8], Supporting Information).

The results indicate that electron beam irradiation can improve the photocatalytic performance of the materials. To investigate the effect of electron beam irradiation on S‐vacancy in the material, electron paramagnetic resonance (EPR) tests were conducted on the samples before and after treatment. The intensity of the EPR signal is directly related to the amount of S‐vacancy in the material. Specifically, an enhancement in the EPR signal usually indicates an increase in the number of S‐vacancy, leading to more unpaired electrons and consequently a stronger EPR signal. This is because S‐vacancy is essentially defects in the lattice, causing changes in the local electronic states. Due to the absence of S atoms, some electrons remain unpaired, exhibiting a local magnetic moment. These unpaired electrons produce characteristic signals in the EPR, and the intensity of these signals reflects the concentration of S‐vacancy. The results demonstrated that the characteristic peaks attributed to S‐vacancy (g = 2.013) were largely not observed in the Bi_2_S_3_@CC sample without electron beam irradiation (Figure S4, Supporting Information). The characteristic peak attributed to S‐vacancy can be observed in Bi_x_/Bi_2‒x_S_y_@CC samples irradiated by electron beam. The characteristic peak of Bi_x_/Bi_2‒x_S_2.89_@CC‐450 is the highest, which indicates that the S‐vacancy content is the highest. Therefore, the high photocatalytic performance of Bi_x_/Bi_2‒x_S_2.89_@CC‐450 may be attributed to the abundance of internal S‐vacancy (i‐S_v_) and Bi^0^ clusters. The presence of Bi^0^ clusters in Bi_x_/Bi_2‒x_S_y_@CC is conducive to the adsorption of CO_2_ on the material surface, resulting in the rapid reduction of CO_2_ to CH_3_OH. Subsequently, the i‐S_v_ is used to adsorb CH_3_OH and accelerate the transfer of gas‐phase products, thereby improving the photoreduction efficiency of CO_2_ by Bi_x_/Bi_2‒x_S_y_@CC.

### 2.3 Stability and Cycling Performance of Prepared Catalysts

**Figure 6:**
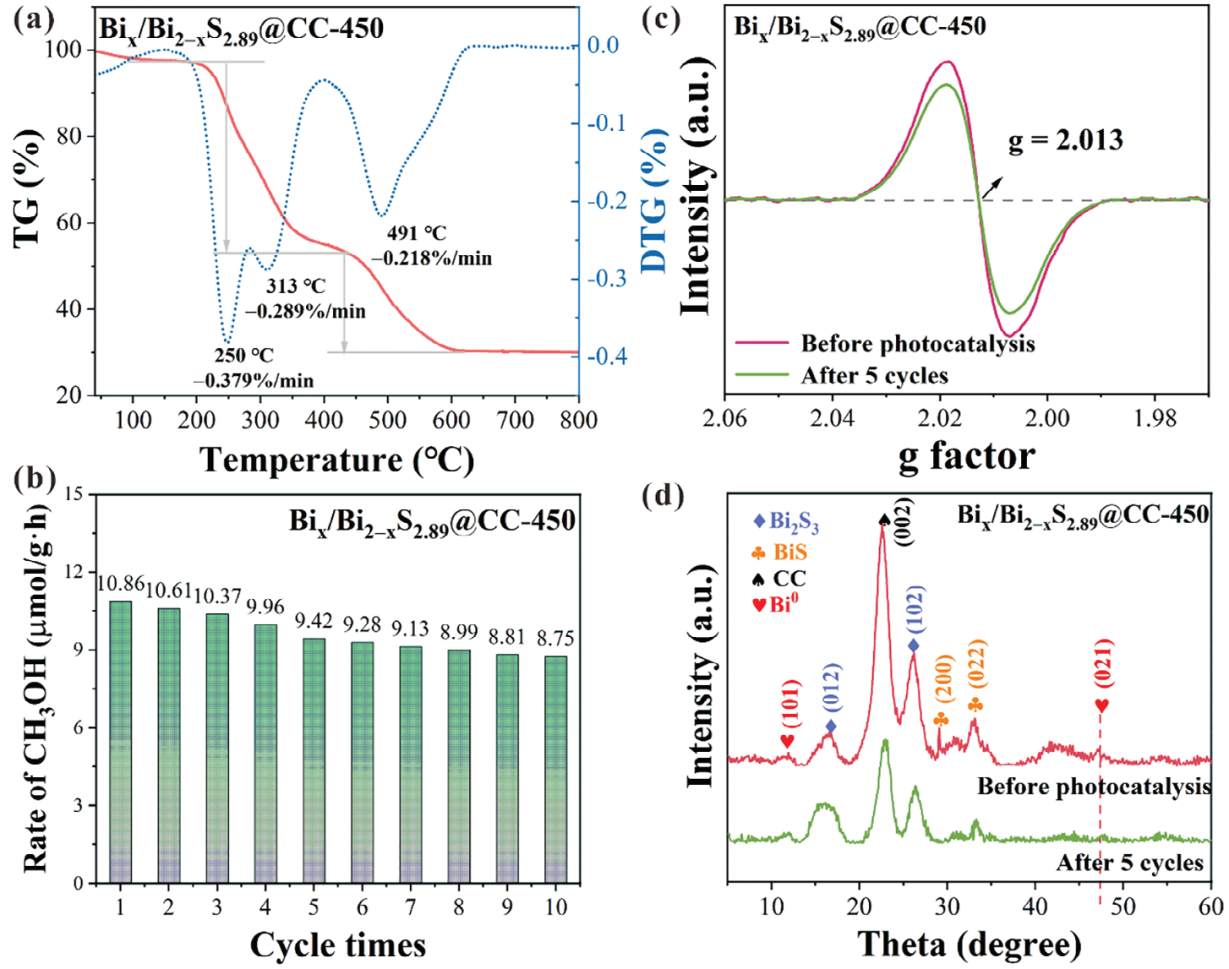
a) TG curve of Bi_x_/Bi_2‒x_S_2.89_@CC‐450. b) EPR characteristic peak of S‐vacancy before and after photocatalysis (five cycles) in Bi_x_/Bi_2‒x_S_2.89_@CC‐450 sample. c) Five cycles of photocatalysis experiment for Bi_x_/Bi_2‒x_S_2.89_@CC‐450 sample. d) XRD spectrum of Bi_x_/Bi_2‒x_S_2.89_@CC‐450 sample before and after photocatalysis (five cycles).

Thermogravimetric analysis was used to evaluate the thermal stability of the prepared samples under a CO_2_ atmosphere. Figure S5 (Supporting Information) shows the TG profile of the Bi_2_S_3_@CC sample. It can be observed that the Bi_2_S_3_@CC sample did not undergo decomposition reactions below 150 °C, and the mass reduction was due to the desorption of water from the sample surface. When the temperature was increased to 475 °C, the mass of the Bi_2_S_3_@CC sample decreased rapidly, suggesting that the cellulose started to undergo thermal decomposition, leading to a decrease in the degree of polymerization. Continuing to increase the temperature to 800 °C, the rate of weight loss of the Bi_2_S_3_@CC sample decreased, suggesting a slow decomposition of the residual solids. Eventually, the residual number of solids in the Bi_2_S_3_@CC sample was ≈28%. Compared to Bi_2_S_3_@CC, the initial decomposition temperature of the Bi_x_/Bi_2‒x_S_2.94_@CC‐150, Bi_x_/Bi_2‒x_S_2.92_@CC‐300, Bi_x_/Bi_2‒x_S_2.89_@CC‐450, and Bi_x_/Bi_2‒x_S_2.98_@CC‐600 samples is higher (Figures S6–S8, Supporting Information; Figure 5a). After experiencing rapid weight loss from 400 to 600 °C, the samples remain stable. This is because electron beam irradiation is a high‐energy treatment that can cause sulfur atoms in the Bi_2_S_3_ lattice to be displaced to form S‐vacancy. The formation of S‐vacancy results in a more locally stable crystal structure. Furthermore, photocatalytic cycle experiments revealed that after ten cycles, the photocatalytic reaction rate of the Bi_x_/Bi_2‒x_S_2.89_@CC‐450 sample for CO₂ reduction decreased to 8.75 µmol·g⁻¹·h⁻¹ (**Figure** **6**b). On the one hand, this decrease is due to some inevitable material loss during repeated washing, as the catalyst used in this study was only 30 mg. On the other hand, as the photocatalytic reaction progresses, S‐vacancy in the sample was affected by the intense light, leading to further migration of S atoms from the lattice or rearrangement of S‐vacancy, which alters the catalytic activity of the material. Additionally, the EPR spectra were observed in Figure 6c. After five reaction cycles, the characteristic EPR peak for S‐vacancy in the Bi_x_/Bi_2‒x_S_2.89_@CC‐450 sample showed a slight decrease, indicating a minor reduction in the concentration of S‐vacancy. Therefore, it can be concluded that the decrease in photocatalytic performance after multiple cycles is primarily due to the reduction in catalyst mass, rather than instability of the prepared sample itself. Figure 6d shows the XRD spectra of the Bi_x_/Bi_2‒x_S_2.89_@CC‐450 samples before and after five cycle experiments. The diffraction peaks of Bi^0^ and BiS in the Bi_x_/Bi_2‒x_S_2.89_@CC‐450 sample decreased after five cycle experiments. It indicates a loss of S‐vacancy and Bi^0^ clusters in Bi_x_/Bi_2‒x_S_2.89_@CC‐450 after the reaction. In conclusion, the prepared samples exhibited good cycling and thermal stability properties.

### 2.4 Photocatalytic Mechanism

The effect of S‐vacancy migration on the reduction of CO_2_ by the Bi_x_/Bi_2‒x_S_y_@CC photocatalyst with bionic chloroplast structure was investigated using the DFT of the Vienna ab initio Simulation Package (VASP).^[^ ^34^ ^]^ The spin‐polarized DFTs were conducted using the VASP. The Perdew–Burke–Ernzerhof generalized‐gradient approximation functional was used to describe the interaction between electrons.^[^ ^35^ ^]^ The energy cutoff was set to 450 eV. The Gamma‐centered k‐point grid was set to be 2  ×  2  ×  1 for all the calculations. The vacuum region was set to be 15 $\dot{A}$ in the z‐direction to prevent the interaction between 2 adjacent surfaces. The convergence threshold for energy and force was set to be less than 10^−5^ eV and 0.02 eV Å^−1^. The DFT‐D3 method is adopted to evaluate the van der Waals interaction.^[^ ^36^, ^37^ ^]^ We calculated the adsorption energy (*E_ads_*) of different species on the surface as follows:

(3) $$E_{ads}=E_{tot}\;-E_{sub}-E_{ads}$$

*E_tot_* and *E_sub_* are the total energies of substrate with and without species adsorption, and *E_ads_* is the energy for the isolated HOCO atom.

**Figure 7:**
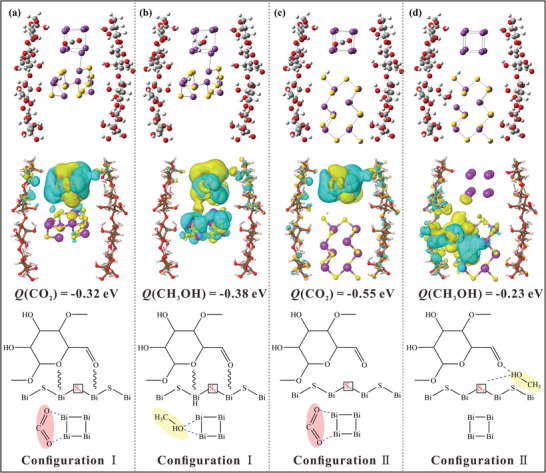
Configuration I adsorbed differential charges of CO_2_ and CH_3_OH. Configuration II adsorbed differential charges of CO_2_ and CH_3_OH.

The catalyst models of Bi_x_/Bi_2‒x_S_y_@CC with b‐S_v_ and i‐S_v_ were constructed according to the structural characteristics of the prepared bionic chloroplast structure photocatalysts. The adsorption configurations of CO_2_ and CH_3_OH and the corresponding charge analysis under the above‐mentioned modes were considered. For ease of identification, the catalysts of the above two structures are named Configuration I and Configuration II, respectively. As shown in **Figure** **7**a,b, the adsorption site of Configurational I for CO_2_ molecules is located on the Bi^0^ cluster, and the adsorption energy is −0.32 eV, suggesting that this adsorption is an exothermic process. The adsorption site of CH_3_OH molecules is also located on the Bi^0^ cluster, and the adsorption energy is greater than that of CO_2_ molecules, which is −0.38 eV (Figure 7b). The yellow and cyan equipotential planes represent charge accumulation (the increase of electron density) and loss (the loss of electron density) in the system, respectively. It can be observed that apparent charge accumulation and loss processes exist between the Bi^0^ cluster and CO_2_/CH_3_OH, suggesting that the Bi_x_/Bi_2‒x_S_y_@CC catalyst with b‐S_v_ is competitive for the adsorption of CO_2_ and CH_3_OH and is more conducive to the adsorption of CH_3_OH. CH_3_OH as the primary catalytic product is adsorbed at the Bi^0^ cluster, which occupies a part of the reaction site for photocatalytic reduction of CO_2_, thus slowing down the continuous progress of the photocatalytic reaction. As shown in Figure 7c, the adsorption site of Configurational II for CO_2_ molecules is still located on the Bi^0^ cluster, and the adsorption energy is more significant than that of Configurational I (−0.55 eV), suggesting that Configurational II has more advantages in adsorption of CO_2_ than Configurational I. Good adsorption capacity provides a solid foundation for the gas‐phase photocatalytic reaction, so that Configurational II is more beneficial to the photoreduction of CO_2_. As shown in Figure 7d, the adsorption site of CH_3_OH by Configurational II is the i‐S_v_ site in Bi_x_/Bi_2‒x_S_y_@CC. In the gas‐phase photocatalytic reaction, the timely transfer of products is exceptionally relevant. Compared with Configurational I, the adsorption site of CH_3_OH was changed in Configurational II, and there was no competitive adsorption relationship between CO_2_ and CH_3_OH. The presence of Bi^0^ clusters in Bi_x_/Bi_2‐x_S_y_@CC is used to adsorb CO_2_ on the material surface, resulting in the rapid reduction of CO_2_ to CH_3_OH. Subsequently, the i‐S_v_ is used to adsorb CH_3_OH and accelerate the transfer of gas‐phase products, thereby improving the photoreduction efficiency of CO_2_ by Bi_x_/Bi_2‒x_S_y_@CC.

**Figure 8:**
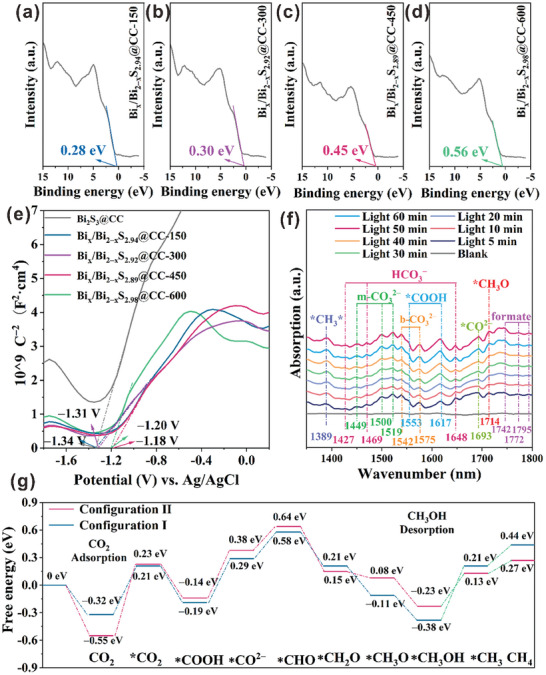
XPS‐VB spectra of a) Bi_x_/Bi_2‒x_S_2.94_@CC‐150, b) Bi_x_/Bi_2‒x_S_2.92_@CC‐300, c) Bi_x_/Bi_2‒x_S_2.89_@CC‐450, and d) Bi_x_/Bi_2‒x_S_2.98_@CC‐600. e) Mott–Schottky curves for each sample. f) In situ diffuse infrared Fourier transform spectra of Bi_x_/Bi_2‒x_S_2.89_@CC‐450 samples at different times. g) Adsorption energy of Configurational I and Configurational II for intermediates in the catalytic conversion of CO_2_.

Figure S9 (Supporting Information) shows the state density maps (DOS) of Configurational I and Configurational II. It can be observed that the valence band (VB) below Fermi level of the two configurations of Bi_x_/Bi_2‒x_S_y_@CC is mainly formed by S orbitals. By contrast, the conduction band (CB) above Fermi level is formed primarily by Bi orbitals. Among them, electrons in the VB of Bi_2‒x_S_y_ can transition to the defect level, thus forming hole carriers. These electrons will be further transferred to the CB of the Bi^0^ cluster, and the transition barrier of electrons to the CB will be reduced while the carrier concentration is increased. The electron conductivity will be significantly improved, thus contributing to the catalytic conversion of CO_2_. In addition, the density of electron states at the Fermi level of Configuration I is higher than that of Configuration II, suggesting that the internal transfer energy of S‐vacancy induced by electron beam irradiation improves the conductivity of the material. The inward migration behavior of the S‐vacancy redistributes the excess electrons to form a narrower band structure that facilitates the electron transition. Figure S10 (Supporting Information) and **Figure** **8**a–d show the XPS‐VB spectra of the prepared samples. It can be observed that the VBs of Bi_2_S_3_@CC, Bi_x_/Bi_2‒x_S_2.94_@CC‐150, Bi_x_/Bi_2‒x_S_2.92_@CC‐300, Bi_x_/Bi_2‒x_S_2.89_@CC‐450, and Bi_x_/Bi_2‒x_S_2.98_@CC‐600 are 0.76, 0.28, 0.30, 0.45, and 0.56 eV, respectively. Figure 8e shows the Mott–Schottky curve of each sample. It can be observed that the prepared samples are N‐type semiconductors, and the flat‐band potentials are −1.34, −1.34, −1.31, −1.18, and −1.20 V. For N‐type semiconductors, the flat‐band potential is positive 0.3 V compared with the CB potential. Therefore, the calculated band conduction potentials of Bi_2_S_3_@CC, Bi_x_/Bi_2‒x_S_2.94_@CC‐150, Bi_x_/Bi_2‒x_S_2.92_@CC‐300, Bi_x_/Bi_2‒x_S_2.89_@CC‐450, and Bi_x_/Bi_2‒x_S_2.98_@CC‐600 are −1.64, −1.64, −1.61, −1.48, and −1.50 eV, respectively. According to VB and CB data, the energy band data diagram of each sample was drawn, as shown in Figure S11 (Supporting Information). The band gaps of Bi_2_S_3_@CC, Bi_x_/Bi_2‒x_S_2.94_@CC‐150, Bi_x_/Bi_2‒x_S_2.92_@CC‐300, Bi_x_/Bi_2‒x_S_2.89_@CC‐450, and Bi_x_/Bi_2‒x_S_2.98_@CC‐600 are 2.40, 1.92, 1.91, 1.93, and 2.06 eV, respectively. Photoluminescence spectroscopy is used to evaluate the electron–hole recombination rate of the prepared sample under light excitation. As shown in Figure S12 (Supporting Information), the maximum excitation wavelength of all samples is ≈570 nm, among which the Bi_2_S_3_@CC sample has the highest PL peak value, corresponding to its faster electron–hole recombination rate. The lowest PL peak value of Bi_x_/Bi_2‒x_S_2.89_@CC‐450 indicates that the electron–hole recombination rate is the slowest, consistent with the highest photocatalytic efficiency. Electrochemical impedance spectroscopy (EIS) is used to study the transfer and recombination processes of photogenerated electrons and holes in various photocatalysts. Typically, a smaller arc radius corresponds to a lower charge transfer resistance, indicating a higher charge separation efficiency for the photocatalyst. As shown in Figure S13 (Supporting Information), the EIS of the Bi_x_/Bi_2‒x_S_2.89_@CC‐450 sample displays the smallest arc radius, suggesting the highest charge separation efficiency. Transient photocurrent testing is used to evaluate the charge separation efficiency and internal electric field strength of photocatalysts. Under illumination, the internal electric field of the photocatalyst can promote the separation of photogenerated electrons and holes, resulting in photocurrent generation. By measuring the photocurrent intensity, the effect of the internal electric field on charge separation can be inferred. Typically, a larger photocurrent indicates a stronger internal electric field and higher charge separation efficiency. As shown in Figure S14 (Supporting Information), the Bi_x_/Bi_2‒x_S_2.89_@CC‐450 sample exhibits higher photocurrent response intensity, indicating the strongest internal electric field. In collisions, the biomimetic chloroplast structure effectively simulates the hierarchical membrane structure and large light‐absorbing surface area of natural chloroplasts, enhancing photon absorption efficiency. Additionally, the biomimetic chloroplast structure facilitates the separation of photogenerated electrons and holes through nanoscale biomimetic design, reducing electron‐hole recombination. Moreover, biomimetic chloroplast structures typically feature a 3D nanonetwork, significantly increasing the specific surface area of the catalyst and providing more active sites. This design enables reactants in the photocatalytic reaction to more easily contact the catalyst surface, thereby improving the reaction rate.

To investigate the reduction process of CO_2_ to CH_3_OH by the photocatalyst prepared, the key intermediates adsorbed on the catalyst surface during the photoreduction reaction were detected by in situ diffuse FT‐IR spectroscopy. As shown in Figure 8f, under dark conditions, Bi_x_/Bi_2‒x_S_2.89_@CC‐450 has almost no apparent characteristic peak between 1350 and 1800 cm^−1^, suggesting no reduction reaction occurs.^[^ ^38^ ^]^ Under irradiation, Bi_x_/Bi_2‒x_S_2.89_@CC‐450 can detect the characteristic vibration peaks of various intermediates adsorbed on the catalyst surface between 1350 and 1800 cm^−1^.^[^ ^39^ ^]^ Specifically, when Bi_x_/Bi_2‒x_S_2.89_@CC‐450 is exposed to a mixed system of CO_2_ and H_2_O, a distinct signal attributed to monodentate carbonate (m‐CO_3_^2−^) can be observed at 1449, 1500, and 1519 cm^−1^.^[^ ^40^ ^]^ A distinct signal attributed to bidentate carbonate (b‐CO_3_^2−^) was observed at 1542 and 1575 cm^−1^, and to bicarbonate (HCO_3_^−^) at 1427, 1469, and 1648 cm^−1^.^[^ ^41^ ^]^ This indicates that the surface of Bi_x_/Bi_2‒x_S_2.89_@CC‐450 can adsorb CO_2_ and H_2_O. The signal peaks at 1553 and 1617 cm^−1^ are directed to ^*^COOH intermediates, and the signal peaks at 1693 cm^−1^ are directed to ^*^CO^2−^ intermediates, which are generally considered key intermediates for carbon dioxide photoreduction.^[^ ^42^ ^]^ The signal peak at 1714 cm^−1^ is attributed to ^*^CH_3_O intermediates. It is the key intermediates in the photochemical conversion of carbon dioxide to methanol.^[^ ^43^ ^]^ In addition, the signal peak at 1389 cm^−1^ is classified as a ^*^CH_3_^*^ intermediate, which is related to the formation of CH_4_.^[^ ^44^ ^]^ The peaks at 1742, 1772, and 1795 cm^−1^ are formatted intermediates, which combine ^*^COOH and ^*^CH_3_ and are eventually depolymerized to CH_3_OH and CH_4_ in a photocatalytic reaction.^[^ ^45^ ^]^ Therefore, the photocatalytic transformation pathway of CO_2_ on Bi_x_/Bi_2‒x_S_2.89_@CC‐450 is as follows:

(4) $$\begin{array}{ccc} & & CO_{2}\to *\mathrm{COOH}\to *CO^{2-}\to *\mathrm{CHO}\to *CH_{2}O\to *CH_{3}O\\ & & \to CH_{3}\mathrm{OH}\to *CH_{3}\to CH_{4} \end{array}$$

In addition, the movement pathway of carbon atoms and intermediate products during the photocatalytic reduction of CO₂ on the Bi_x_/Bi_₂‒x_S_2.89_@CC‐450 catalyst was tracked by C isotope experiment. As shown in Figure S15 (Supporting Information), mass spectrometry (MS) signals of ¹^3^CH, ¹^3^CH₂, ¹^3^CH₃, H₂O, ¹^3^CHO, ¹^3^CH₂O, ¹^3^CH₃O, ¹^3^CH₃OH, CO₂, and ¹^3^COOH were clearly detected during the isotope experiment. This further confirms that methanol is formed from the photocatalytic reduction of CO₂ on Bi_x_/Bi_2‒x_S_2.89_@CC‐450.

To further understand the reasons for the different photocatalytic activities of Configurational I and Configurational II on CO_2_, DFT was applied to explore the adsorption energies of the two on intermediates during the catalytic CO_2_ conversion process (Figure 8g). First, Configurational I and Configurational II adsorb CO_2_ and generate ^*^CO_2_ intermediates on their surfaces. Subsequently, the ^*^CO_2_ intermediates are hydrogenated to produce ^*^COOH intermediates, which are further converted to ^*^CO^2−^ and ^*^OH intermediates. ^*^CO^2−^ intermediate will be gradually hydrogenated until CH_3_OH is formed. Among them, a small part of CH_3_OH is converted and hydrogenated to form CH_4_. Notably, the adsorption energy of the two kinds of adsorption intermediates is quite different. Specifically, the adsorption energy of Configurational II for CO_2_ is −0.55 eV, which is more negative than that of Configurational I (−0.32 eV), suggesting that the adsorption of CO_2_ by both configurations is an exothermic process, and Configurational II has more advantages in the adsorption of CO_2_. The adsorption energy of Configurational I and Configurational II to ^*^CO_2_ intermediates is 0.21 and 0.23 eV, respectively, which is an endothermic process. In addition, the adsorption energies of Configurational I and Configurational II to ^*^COOH intermediates are negative (−0.19 and −0.14 eV, respectively), suggesting that this process is thermodynamically spontaneous. Subsequently, the ^*^COOH intermediate is decomposed and hydrogenated several times to obtain the ^*^CH_3_O intermediate, which is one of the key steps to obtain the product CH_3_OH. Notably, the adsorption energy of Configurational I for ^*^CH_3_O intermediate is −0.11 eV, which is more negative than that of Configurational II (0.08 eV), suggesting that Configurational I is more conducive to the adsorption of ^*^CH_3_O intermediate. In short, Bi_x_/Bi_2‒x_S_y_@CC materials with i‐S_v_ are more conducive to the adsorption of reactant CO_2_ than to the adsorption of reaction product methanol. The structural model diagrams of the intermediates adsorbed in configuration II was showed Figure S16 (Supporting Information). On the one hand, the S‐vacancy migrates from the interface to the interior of the material, so that the active site is no longer concentrated on the surface, but distributed in the internal structure of the catalyst. This migration helps to reduce the recombination of photoelectrons and holes, allowing the electrons to participate more effectively in the reduction reaction, and thus preferring to reduce CO_2_ to CH_3_ over other products. This migration helps reduce the recombination of photogenerated electrons and holes, allowing electrons to more effectively participate in the reduction reaction, favoring the conversion of CO₂ to CH_3_OH over other products. As the S‐vacancy migrate inward, CO_2_ and intermediates (such as COOH^*^) are more likely to remain at the internal adsorption sites and, through a series of protonation steps, generate CH_3_OH. The redistribution of sites due to S‐vacancy migration helps separate products from reactants during the reaction, reducing competitive adsorption between reactants and products, thereby increasing the selectivity for CH₃OH. On the other hand, the position of internal S‐vacancy provides a shorter transfer path for photogenerated electrons, avoiding electron‐hole recombination caused by interface defects, which enhances the utilization efficiency of photogenerated electrons. Electrons primarily concentrate near the CO_2_ adsorption sites, facilitating the more efficient reduction of CO_2_ to CH_3_OH. The inward migration and distribution of S‐vacancy locally alter the electronic structure, narrowing the bandgap of the material, further promoting electron transitions and reduction reactions. The redistributed S‐vacancy allocate excess electrons, thereby enhancing the selectivity of the CO_2_ reduction reaction toward CH_3_OH.

**Figure 9:**
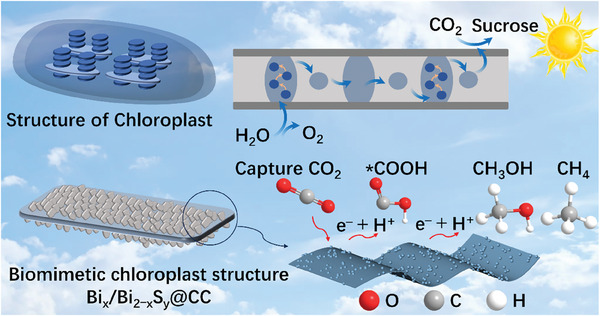
Mechanism process.

In conclusion, combined with experimental data and theoretical calculations, the mechanism of Bi_x_/Bi_2‒x_S_y_@CC for efficient selective photocatalytic CO_2_ to CH_3_OH was rationally proposed (**Figure** **9**). First, the macroscopical mechanism is as follows. Electron beam irradiation results in forming the b‐S_v_ and Bi° clusters in Bi_x_/Bi_2‒x_S_y_@CC, and the adsorption sites related to reactant (CO_2_) and product (CH_3_OH) are at the Bi^0^ cluster. Notably, there is a competitive adsorption relationship between the reactants and the products in the structure, which is more conducive to the adsorption of the products. With the increase in electron beam irradiation intensity, the boundary S‐vacancy (b‐S_v_) in Bi_2_S_3_@CC undergoes inward migration and generates Bi^0^ clusters in their original positions. Here, the adsorption site related to the reactant (CO_2_) is located at the Bi° cluster. By contrast, the adsorption site pertaining to the product (CH_3_OH) is located at the i‐S_v_. Notably, there is no competitive adsorption relationship between reactants and products in this structure. The presence of Bi^0^ clusters in Bi_x_/Bi_2‒x_S_y_@CC is conducive to the adsorption of CO_2_ on the material surface, resulting in the rapid reduction of CO_2_ to CH_3_OH. Subsequently, CH_3_OH is rapidly transferred to i‐S_v_ and releases more photoreactive sites, ultimately promoting the continuation of the photoreduction reaction. Second, the microcosmic mechanism is as follows. In short, photoexcitation causes electrons in the VB of Bi_x_/Bi_2‒x_S_y_@CC to transfer to the CB, thereby leaving photogenerated holes (h^+^). The directional inward migration of S‐vacancy and the formation of Bi^0^ clusters in situ in Bi_x_/Bi_2‒x_S_y_@CC photocatalysts redistribute the excess electrons in the photoreaction to form a narrower band structure, which facilitates the electron transition and promotes the photoreduction reaction. Bi_x_/Bi_2‒x_S_y_@CC adsorbs CO_2_ and produces COOH^*^ intermediates under the action of photogenic electrons, and COOH^*^ intermediates are protonated continuously to produce CH_3_OH.

## 3 Conclusion

In summary, stable Bi_2_S_3_@CC photocatalysts based on CC were constructed to probe the effect of the directional migration of S‐vacancy on the separation of photogenerated carriers and the electron transition of semiconductors. S‐vacancy was induced in Bi_2_S_3_@CC by electron beam irradiation to obtain Bi_x_/Bi_2‒x_S_y_@CC photocatalysts with biomimetic chloroplast structures. This Bi_x_/Bi_2‒x_S_y_@CC photocatalyst exhibits good efficiency and high selectivity in the photoreduction of CO_2_ to methanol. Electron beam irradiation induces the conversion of boundary S‐vacancy (b‐S_v_) in Bi_x_/Bi_2‒x_S_y_@CC into i‐S_v_ after directional inward migration and generates the Bi^0^ clusters at their original positions. By contrast, the in‐migration behavior of boundary sulfur vacancy (b‐S_v_) in Bi_x_/Bi_2‒x_S_y_@CC will promote the redistribution of electrons in the semiconductor, which will improve the separation efficiency of the photogenerated carrier and the transition efficiency of the electron. The presence of Bi^0^ clusters in Bi_x_/Bi_2‒x_S_y_@CC is conducive to the adsorption of CO_2_ on the material surface, resulting in the rapid reduction of CO_2_ to CH_3_OH. Subsequently, CH_3_OH is rapidly transferred to i‐S_v_ and releases more photoreactive sites, ultimately promoting the continuation of the photoreduction reaction. The Bi_x_/Bi_2‒x_S_y_@CC photocatalyst with bionic chloroplast structure can not only efficiently enrich CO_2_ and conduct photoreduction reactions at room temperature and visible light, thus realizing the controllable conversion of CO_2_ and photoreduction products at the gas–liquid–solid three‐phase interface. This study clarified the law and mechanism of the effect of S‐vacancy on the photocatalytic reduction of CO_2_ to CH_3_OH. It provided another idea for the application of cellulose‐based bionic chloroplast structure photocatalysts in CO_2_ capture and conversion.

## 4 Experimental Section

### Preparation of Samples

After the wax component was removed in a toluene–ethanol‐mixed solution, the bamboo powder was dignified with a 3% NaClO_2_ solution in an 85 °C water bath until it turned completely white. Subsequently, the hemicellulose was further removed by adding an 8% NaOH solution to obtain bamboo cellulose. Furthermore, 10 g of bamboo cellulose was added to deionized water and stirred vigorously for 30 min. Subsequently, 0.4 g NaBr and 0.08 g 2,2,6,6‐tetramethylpiperidinyl‐1‐oxide (TEMPO) were added, and the NaOCl solution was dripped at room temperature. The pH of the mixture was adjusted to 10 with NaOH solution, the oxidation time was 6 h, and then the mixture was washed to assume neutrality. After five cycles using a high‐pressure homogenizer, the mixture was placed in a regenerated cellulose dialysis tube, and the deionized water was dialyzed for 5 days to obtain carboxylic cellulose (CC).

Bi_2_S_3_@CC was prepared by a coprecipitation method. First, 2.72 g of bismuth nitrate and CC were added to 40 mL of deionized water and stirred for 30 min in a water bath at 60 °C to obtain solution A. Subsequently, 1.92 g of Na_2_S·9H_2_O was added to 40 mL of deionized water to obtain solution B. Solution B was added dropwise to solution A to obtain Bi_2_S_3_@CC. Solution B was washed several times with deionized water to remove excess cations from the system and placed in a freeze dryer for several days to obtain a black solid.

The black solid obtained above was divided into four parts, and the Bi_x_/Bi_2‒x_S_y_@CC bionic chloroplast material was obtained by irradiation with an intensity of 150, 300, 450, and 600 KGy, respectively. The chemical constituents of the four samples were characterized by X‐ray photoelectron spectroscopy (XPS) and named Bi_x_/Bi_2‒x_S_2.94_@CC‐150, Bi_x_/Bi_2‒x_S_2.92_@CC‐300, Bi_x_/Bi_2‒x_S_2.89_@CC‐450, and Bi_x_/Bi_2‒x_S_2.98_@CC‐600, respectively.

### Characterization

A field‐emission scanning electron microscope (SUPRA 55 SAPPHIRE) manufactured in Germany was used to study the morphological characteristics of the samples. A transmission electron microscope (JEOL JEM 2100F) manufactured in Japan was used to observe the fine structure inside the sample. The thermal stability of various materials was determined by the thermogravimetry analysis (TG, METTLER TOLEDO, TGA/DSC1/1100LF) produced by Switzerland. XPS (Thermo ESCALAB 250XI) was used to analyze the chemical valence states of the elements in the sample. An X‐ray diffractometer (XRD, X'Pert PRO MPD) was used to characterize the crystal structure and its variation. Fourier transform infrared spectroscopy (FT‐IR, Nicolet iS 10) was used to study the structure of molecules and the adsorption dynamics on the surface of photocatalytic catalysts. Paramagnetic resonance spectroscopy (EPR, Bruker EMXplus) was used to capture the S‐vacancy in the prepared sample. The electrochemical workstation was used to obtain the Mott–Schottky curve, transient photocurrent curve, and electrochemical impedance spectrum of the sample. Among them, the reference electrode was Ag/AgCl, and the opposite electrode was a platinum sheet electrode. A steady‐state/transient fluorescence spectrometer was used to obtain the photoluminescence spectrum (PL, Edinburgh FLS980) of the sample.

### CO_2_ Photocatalysis Experiment

The photocatalytic CO_2_ reduction was conducted at about room temperature of 25 °C in a sealed system. A 300 W Xe lamp (PLS‐SXE300) was employed as the light source. For the photocatalytic experiments, 30 mg of catalyst was added to 10 mL of deionized water, and a suspension was obtained after the mixture was incubated for 30 min. The reactor was sealed after the suspension was added and purged with CO_2_ to remove the dissolved oxygen. Subsequently, the photoreaction system was purged with CO_2_ gas for ≈120 min to reach the adsorption/desorption equilibrium of CO_2_ and the catalyst surface. The obtained gaseous products were analyzed using a gas chromatograph (7900 A, Techcomp) equipped with a flame ionization detector and a thermal conductivity detector via an automated gas valve with helium as the carrier gas. By contrast, the liquid products were measured by GC–MS (GC7890B‐5977A MSD).

## Conflict of Interest

The authors declare no conflict of interest.

## Author Contributions

W.W. conceived and designed the experiment. Y.W. and W.W. as supervisors jointly provided funding support and revisions to the manuscript. J.W. did the experiments and wrote the manuscript. Y.D. and Z.Z. helped with the experiment. H.W. provided the electron beam irradiation equipment. All authors discussed the findings and commented on the manuscripts.

## Supporting information



Supporting Information

## Data Availability

The data that support the findings of this study are available in the supplementary material of this article.
