# Supplementary material for: Directed Inward Migration of S‐Vacancy in Bi2S3 QDs for Selective Photocatalytic CO2 to CH3OH
Source: Adv Sci (Weinh). 2025 Jan 9;12(8):2406925. doi: 10.1002/advs.202406925 (PMC11848541; doi:10.1002/advs.202406925)
Supplement: Supplementary file 1 — Supporting Information [file ADVS-12-2406925-s001.docx]

Directed inward migration of S-vacancy in Bi_2_S_3_ QDs for selective photocatalytic CO_2_ to CH_3_OH

Jing Wang, Wenlei Wang*, Yao Deng, Zhen Zhang, Hui Wang, Yiqiang Wu*

J. Wang, Z. Zhang, H. Wang, Y.Q. Wu

College of Materials Science and Engineering

National and Local Joint Engineering Research Center for Green Processing Technology of Agricultural and Forestry Biomass

Central South University of Forestry and Technology

Changsha 410004, China

W.L. Wang, Y. Deng

College of Chemistry and Chemical Engineering

National Forestry and Grassland Administration Bioethanol Research Center, College of Chemistry and Chemical Engineering

Central South University of Forestry and Technology

Changsha 410004, China

**Correspondence**

Yiqiang Wu

Email: [wuyiqiang@csuft.edu.cn](mailto:wuyiqiang@csuft.edu.cn), [wuyq0506@126.com](mailto:wuyq0506@126.com)

Wenlei Wang

Email: [wenlei_wang@csuft.edu.cn](mailto:wenlei_wang@csuft.edu.cn), [wenlei_wang@hotmail.cn](mailto:wenlei_wang@hotmail.cn)


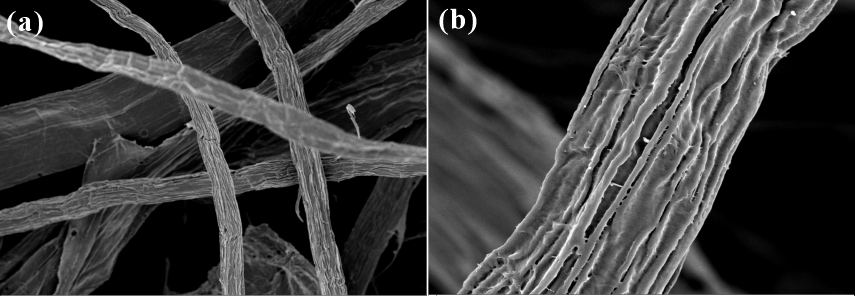


**Figure S1.** SEM image of Bi_2_S_3_@CC sample.


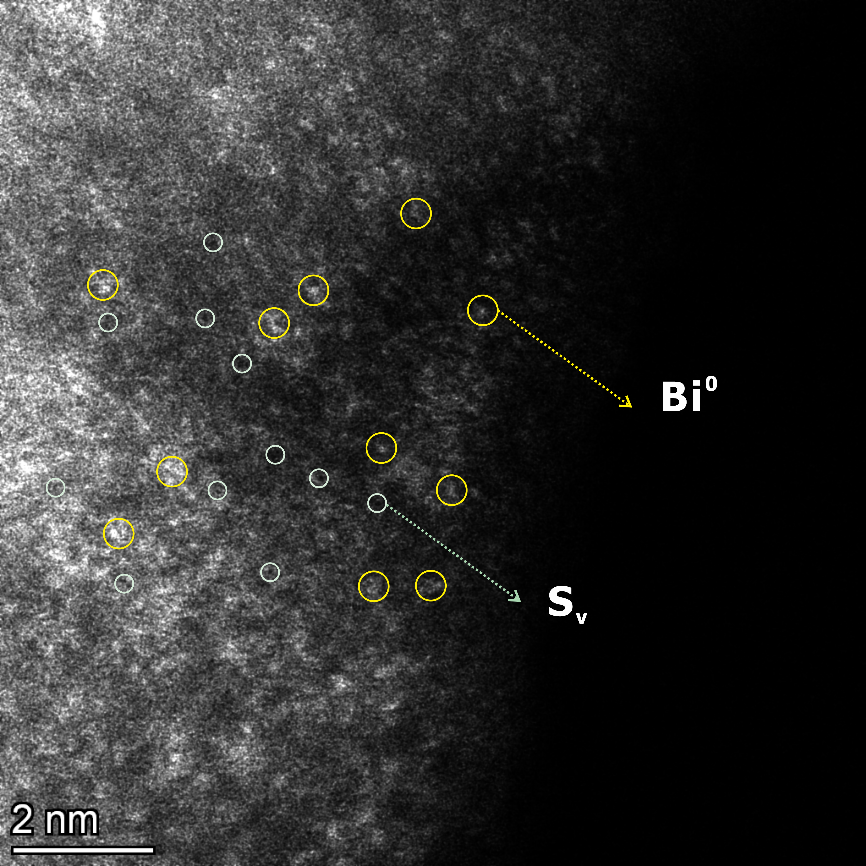


**Figure S2.** HAADF-STEM image of Bi_x_/Bi_2‒x_S_2.89_@CC-450.





**Figure S3.** XPS spectra of C elements for each sample.





**Figure S4.** EPR captures the S-vacancy.





**Figure S5.** TG curve of Bi_2_S_3_@CC sample.





**Figure S6.** TG curve of Bi_x_/Bi_2‒x_S_2.94_@CC-150 sample.





**Figure S7.** TG curve of Bi_x_/Bi_2‒x_S_2.92_@CC-300 sample.





**Figure S8.** TG curve of Bi_x_/Bi_2‒x_S_2.98_@CC-600 sample.





**Figure S9.** State density maps of Configuration I and Configuration II.





**Figure S10.** XPS-VB spectra of Bi_2_S_3_@CC samples.





**Figure S11.** The band gaps of each sample.





**Figure S12.** The photoluminescence (PL) spectroscopy of each sample.





**Figure S13.** The electrochemical impedance spectroscopy (EIS) of each sample.





**Figure S14.** The transient photocurrent response spectrum of each sample.





**Figure S15.** MS signals obtained in photoreduction of ^13^CO_2_ with H_2_O on Bi_0.17_/Bi_2_S_2.89_@MC-450.


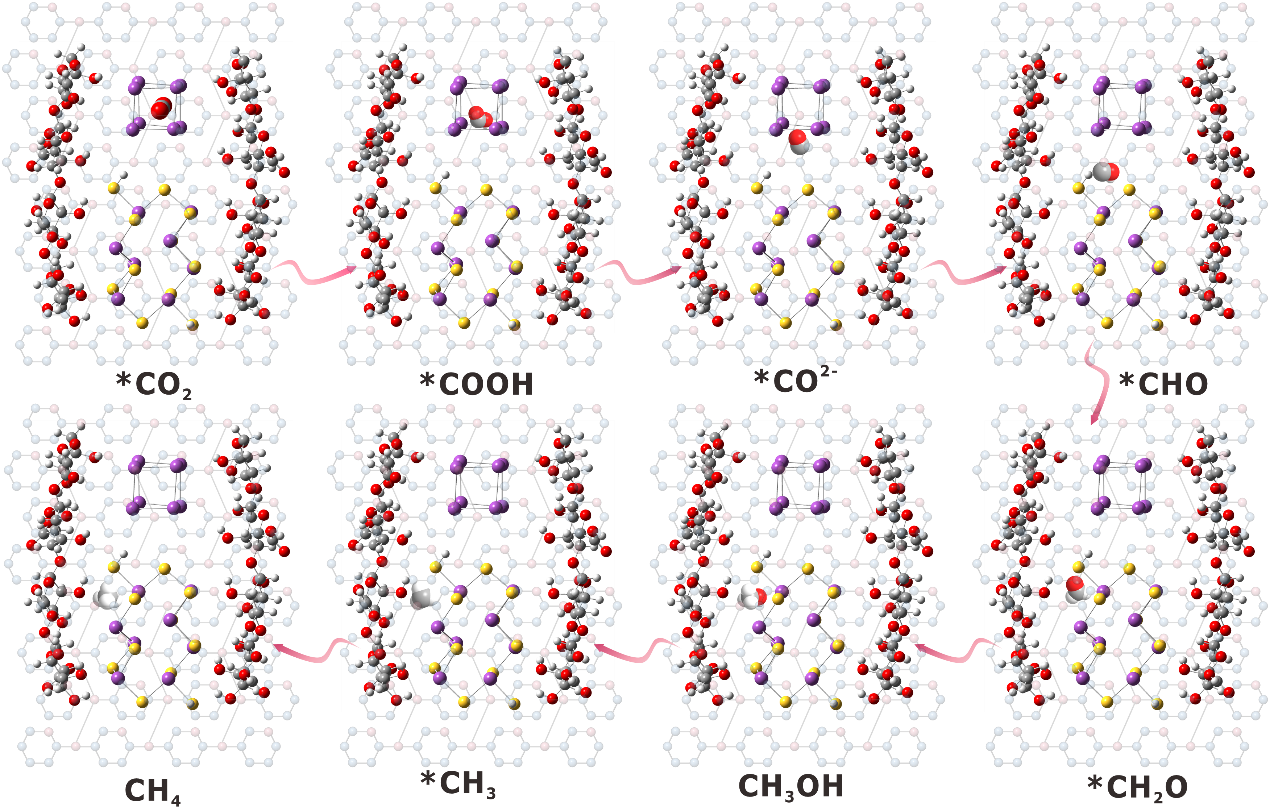


**Figure S16.** Structural model of intermediates in the catalytic conversion of CO_2_ for Configurational II. **Table S1.** Content of corresponding elements in each sample.

|  |  | Percentage (At%) | | | | |
| --- | --- | --- | --- | --- | --- | --- |
| Sample | EBI Intensity (KGy) | Bi^0^ | Bi^n+^ | S | C | O |
| Bi_2_S_3_@MC | 0 | ‒ | 18.63 | 26.79 | 38.11 | 16.47 |
| Bi_0.12_/Bi_2_S_2.94_@MC-150 | 150 | 1.08 | 17.80 | 26.17 | 37.65 | 17.30 |
| Bi_0.13_/Bi_2_S_2.92_@MC-300 | 300 | 1.14 | 17.46 | 25.50 | 37.81 | 18.09 |
| Bi_0.17_/Bi_2_S_2.89_@MC-450 | 450 | 1.42 | 17.28 | 24.84 | 38.46 | 18.00 |
| Bi_0.14_/Bi_2_S_2.98_@MC-500 | 600 | 1.29 | 17.85 | 26.63 | 38.98 | 15.25 |

Table. S2. Comparison of catalyst performance reported in other literature.

| Catalysts | Catalyst dosage (h) | Light source | Reaction time  (h) | Main product | Yields (μmol·g^−1^·h^−1^) | AQE (%) | Ref. |
| --- | --- | --- | --- | --- | --- | --- | --- |
| NAL@MRF | 50 | 300 W Xe lamp | 4 | CH_3_OH | 31.41 | 2.4800 | [S1] |
| CeO_2_@Bi_2_MoO_6_ | 20 | 300 W Xe lamp | 4 | CH_3_OH | 4.31 | 0.1300 | [S2] |
| 8%Cu-SrTiO_3_ | 10 | 300 W Xe lamp | 4 | CH_3_OH | 8.08 | 12.7724 | [S3] |
| La_0.9_FeO_3−δ_ | 20 | 300 W Xe lamp | - | CH_3_OH | 5.30 | 0.1677 | [S4] |
| AUiO@CN | 10 | 300 W Xe lamp | 7 | CH_3_OH | 9.32 | 0.0986 | [S5] |
| 3%MnO_x_/BiVO_4_/Bi_2_S_3_ | 30 | 300 W Xe lamp | 6 | CH_3_OH | 13.75 | 0.0652 | [S6] |
| Bi_4_Ti_3_O_12_ | 20 | 300 W Xe lamp | 4 | CH_3_OH | 1.40 | 0.0443 | [S7] |
| XCN@M | 100 | 300 W Xe lamp | 5 | CH_3_OH | 0.99 | 0.0179 | [S8] |
| Bi_x_/Bi_2‒x_S_y_@CC | 30 | 300 W Xe lamp | 6 | CH_3_OH | 10.74 | 0.0509 | This work |

**References**

[S1] R.N. Wang, M.J. Zhang, S.L. Zhang, J.Z. Zheng, Y.Q. Zeng, Y. Yang, J. Ding, X. Wu, Q. Zhong, Self-supporting triphase photocatalytic CO_2_ reduction to CH_3_OH on controllable core−shell structure with tunable interfacial wettability, *ACS Nano* **2023**, 17, 24363−24373.

[S2] Y.J. Xie, C.H. Qiu, L. Wang, Y.X. Wang, J. Zhang, J.F. Zhang, H. Wan, G.F. Guan, Microwave-assisted fabrication of 1D/2D CeO_2_/Bi_2_MoO_6_ heterojunction for efficient photocatalytic CO_2_ reduction to CH_3_OH, *Ceram. Int.* **2024**, 50, 25161‒25169.

[S3] X.Q. Guo, C.H. Qiu, Z.Q. Zhang, J. Zhang, L. Wang, J. Ding, J.F. Zhang, H. Wan, G.F. Guan, Coaxial electrospinning prepared Cu species loaded SrTiO_3_ for efficient photocatalytic reduction of CO_2_ to CH_3_OH, *J. Environ. Chem. Eng.* **2024**, 12, 111990.

[S4] C.H. Qiu, L. Wang, R.J. Chen, J. Zhang, J. Ding, J.F. Zhang, H. Wan, G.F. Guan, Insight of the state for deliberately introduced a‑site defect in nanofibrous LaFeO_3_ for boosting artificial photosynthesis of CH_3_OH, *ACS Appl. Mater. Interfaces* **2023**, 15, 56945−56956.

[S5] H.W. Guo, T.X. Zhang, W.H. Ma, S.W. Cheng, J. Ding, Q. Zhong, S. Kawi, Construction of sandwich-like Ag/UiO-66@g-C_3_N_4_ Z-scheme ternary heterojunction for photocatalytic CO_2_ conversion to CH_3_OH and CO, *Fuel* **2023**, 344, 127911.

[S6] M.Y. Wang, S.M. Zeng, A.R. Woldu, L.S. Hu, BiVO_4_/Bi_2_S_3_ Z-scheme heterojunction with MnO_x_ as a cocatalyst for efficient photocatalytic CO_2_ conversion to methanol by pure water, *Nano Energy* **2022**, 104, 107925.

[S7] Y.X. Wang, C.H. Qiu, Y.J. Xie, L. Wang, J. Ding, J.F. Zhang, H. Wan, G.F. Guan, Intentionally Introducing Oxygen Vacancies and Ti^3+^ Defects on the Surface of Bi_4_Ti_3_O_12_ Nanosheets for Promoting the Photoreduction of CO_2_ to CH_3_OH, *ACS Appl. Nano Mater.* **2024**, 7, 3012−3023.

[S8] J. Ding, Q.L. Tang, Y.H. Fu, Y.L. Zhang, J.M. Hu, T. Li, Q. Zhong, M.H. Fan, H.H. Kung, Core−shell covalently linked graphitic carbon nitride−melamine−resorcinol−formaldehyde microsphere polymers for efficient photocatalytic CO_2_ reduction to methanol, *J. Am. Chem. Soc.* **2022**, 144, 9576−9585.
